# RNA *N*6-methyladenosine: a promising molecular target in metabolic diseases

**DOI:** 10.1186/s13578-020-00385-4

**Published:** 2020-02-21

**Authors:** Yan Li, Jiawen Wang, Chunyan Huang, Meng Shen, Huakui Zhan, Keyang Xu

**Affiliations:** 1 Hospital of Chengdu University of Traditional Chinese Medicine, Chengdu, 610072 Sichuan China; 2 Houjie Hospital of Dongguan, Dongguan, 523945 Guangdong China; 3 Chengdu Tumor Hospital, Chengdu, 610041 Sichuan China; 4 Hangzhou Xixi Hospital Affiliated to Zhejiang Chinese Medical University, Hangzhou, 310023 Zhejiang China

**Keywords:** *N*6-methyladenosine, Methylation, Methyltransferase, Demethylase, Adipogenesis, Metabolic diseases

## Abstract

*N*6-methyladenosine is a prevalent and abundant transcriptome modification, and its methylation regulates the various aspects of RNAs, including transcription, translation, processing and metabolism. The methylation of *N*6-methyladenosine is highly associated with numerous cellular processes, which plays important roles in the development of physiological process and diseases. The high prevalence of metabolic diseases poses a serious threat to human health, but its pathological mechanisms remain poorly understood. Recent studies have reported that the progression of metabolic diseases is closely related to the expression of RNA *N*6-methyladenosine modification. In this review, we aim to summarize the biological and clinical significance of RNA *N*6-methyladenosine modification in metabolic diseases, including obesity, type 2 diabetes, non-alcoholic fatty liver disease, hypertension, cardiovascular diseases, osteoporosis and immune-related metabolic diseases.

## Introduction

A total of 160 RNA modifications have been reported to participate in life activities and diseases progress, especially methylation [1]. In eukaryotic mRNA, there are several identified methylation modifications, such as *N*(7)-methylguanosine, *N*(6)-methyl-2′-*O*-methyladenosine, 2′-*O*-methylation, *N*(6)-methyladenosine (m^6^A) and 5-methylcytosine (m^5^C) [2]. Among them, m^6^A has been considered as the most abundant internal modification, since it was discovered from methylated nucleosides in mRNA of Novikoff hepatoma cells in the early 1970s [3]. m^6^A is enriched in stop codon and 3′ untranslated terminal region (UTR) and translates near 5′ UTR in a cap-independent manner [4–6], thereby regulating RNA transcription, translation, processing and metabolism [5, 6]. The process of m^6^A modification is reversible and can be regulated by three homologous factors jargonized as ‘writers’, ‘erasers’ and ‘readers’ [7, 8]. For example, ‘Writers’ are categorized as the components of that catalyze the formation of m^6^A methylation [7, 9]; ‘Erasers’ play an important role in m^6^A modification for their demethylated functions [10, 11]; ‘Readers’ are a group of molecules which can decode m^6^A methylation and generate functional signals [12, 13]. So far, m^6^A has been found not only in mRNAs, but also in a variety of non-coding RNAs including rRNA, tRNA, snRNA, miRNA, and lncRNA [14, 15]. For example, m^6^A methyltransferase-like 3 (METTL3) interacts with the microprocessor protein DGCR8 and modulates miR-873-5p mature process positively [16]. The expression of m^6^A demethylase fat mass and obesity-associated protein (FTO) can influence the steady state level of various miRNAs, including increased expression of hsa-miR-6505-5p, hsa-miR-651-5p and hsa-miR-493-5p, and decreased expression of hsa-miR-7-5p, hsa-miR-92a-1-5p and hsa-miR-6769a-3p [15]. In addition, m^6^A modification of lncRNAs can induce the proliferation, metastasis and apoptosis of cancer cells [17]. For example, alkB homolog 5 (ALKBH5) inhibits pancreatic cancer motility by demethylating lncRNA KCNK15-AS1 [18], and METTL16 can methylate diverse cellular RNAs in human embryonic kidney 293 cells, consisting of 8 pre-mRNAs, 355mRNAs, 68 lncRNAs and other type of RNAs [19].

m^6^A RNA modification is a widespread and reversible process, which is highly associated with multiple diseases such as metabolic diseases (MDs), infertility, virus infection and cancers [20–23]. In this review, we aim to summarize the biological features and therapeutic potentials of m^6^A modifications in MDs.

## Metabolic diseases

MDs refer to the pathological results of metabolic disorders of proteins, fats, carbohydrates and other substances in the human body [24], including obesity, type 2 diabetes (T2D), non-alcoholic fatty liver disease (NAFLD), hypertension, osteoporosis, chronic kidney disease, cardiovascular disease and other related metabolic disorders [25]. Currently, there are over 1.9 billion adults and 340 million children and adolescents with overweight or obese [26], more than 415 million people with diabetes [27], and 6–35% (median 20%) of population with NAFLD [28] around the world. In the past decades, the various treatments were used to prevent and treat the aforementioned MDs but they are still limited [29]. For diabetes, the long term treatment is insufficient for controlling blood glucose by daily medicines take like metformin or subcutaneous injection of insulin, as blood glucose is easy fluctuated by the intake of food and physical activity [30]. For the treatment of NAFLD, although lifestyle modification, vitamin E, and clinical surgery as main methods are commonly used, there is no effective medicine to prevent the pathological development of it [31]. Recently, m^6^A RNA modification has been found to be involved in the development of MDs [32–34] (Table 1). Therefore, m^6^A modification might be potential targets for the therapy and early diagnosis of MDs.

**Table 1:** The functions of RNA m^6^A methylation in metabolic diseases

	m^6^A Regulators	Functions	Refs
T2D	FTO	Promoting the mRNA expression of FOXO1, G6PC, and DGAT2, which are associated with glucose and lipid metabolism	[32]
	METTL3	Inhibiting hepatic insulin sensitivity via *N*6-methylation of FASN mRNA and promoting fatty acid metabolism	[69]
		Upregulating insulin/IGF1–AKT–PDX1 pathway in human β-cells	[71]
	METTL14	Decreasing cell death and the changes of cell differentiation of β-cells, increasing β-cell mass and insulin secretion	[70]
		Upregulating insulin/IGF1–AKT–PDX1 pathway in human β-cells	[71]
Obesity	FTO	Promoting adipogenesis by inhibiting the Wnt/β-catenin signaling pathway	[86]
		Promoting autophagy and adipogenesis via increasing the expression of ATG5 and ATG7	[87]
		Promoting adipocyte proliferation via enhancing the expression of the pro-adipogenic short isoform of RUNX1	[77]
	WTAP	Suppressing adipogenesis by promoting cell cycle transition in mitotic clonal expansion	[89]
	METTL3	Suppressing adipogenesis by promoting cell cycle transition in mitotic clonal expansion	[89]
		Inhibiting adipogenesis via the depletion of ZFP217 and CCND1	[92]
	METTL14	Suppressing adipogenesis by promoting cell cycle transition in mitotic clonal expansion	[89]
	YTHDF2	Inhibiting autophagy and adipogenesis by decreasing protein expression of ATG5 and ATG7 and shortening the lifespan of their m^6^A-modified mRNAs	[87]
		Suppressing adipogenesis by increasing m^6^A methylation of CCNA2 and CDK2 and reversing the methylation effect of FTO on CCNA2 and CDK2	[90, 91]
		Inhibiting adipogenesis via the downregulation of CCND1	[92]
NAFLD	FTO	Down-regulating mitochondrial content and up-regulating TG deposition	[101]
		Promoting hepatic fat accumulation by increasing the expression of lipogenic genes, including FASN, SCD and MOGAT1, and intracellular TG level in HepG2 cells	[101]
		Increasing oxidative stress and lipid deposition	[99]
	YTHDF2	Increasing lipid accumulation by decreasing both PPARα mRNA lifetime and expression	[105]
	METTL3	Increasing lipid accumulation by decreasing both PPARα mRNA lifetime and expression	[105]
Hypertension	m^6^A-SNPs	EncodIing β1-adrenoreceptor, a hypertension-susceptibility candidate gene	[108, 109]
		Altering BP-related gene expression, mRNA stability and homeostasis	[110]
Cardiovascular diseases	FTO	Decreasing fibrosis and enhancing angiogenesis in mouse models of myocardial infarction	[111]
	METTL3	Driving cardiomyocyte hypertrophy by catalyzing methylation of m^6^A on certain subsets of mRNAs	[112]
		Decreasing eccentric cardiomyocyte remodeling and dysfunction	[112]
		Inhibiting cellular autophagic flux and promoting apoptosis in hypoxia/reoxygenation-treated cardiomyocytes	[113]
Osteoporosis	METTL3	Inhibiting adipogenesis and adipogenic differentiation via JAK1/STAT5/C/EBPβ pathway in bone marrow stem cells	[119]
		Inhibiting osteoporosis pathological phenotypes, consisting of decreased bone mass and increased marrow adiposity via PTH/PTH1R signaling axis	[118]
	FTO	Promoting the differentiation of adipocyte and osteoblast by upregulating GDF11–FTO–PPARγ signalling way	[116]
		Enhancing the stability of mRNA of proteins which function to protect osteoblasts from genotoxic damage through Hspa1a–NF-κB signaling way	[120]
Immune-related MDs	ALKBH5	Expressing highly in organs enriched in immune cells with frequent immune reactions	[10, 123]
	METTL3	Stimulating T cell activation and the development of T lymphocytes in the thymus by regulating the translation of CD40, CD80 and TLR4 signaling adaptor TIRAP transcripts in dendritic cells	[124, 125]
		Maintaining T cell homeostasis and differentiation by targeting the IL-7/STAT5/SOCS pathways	[126]

m^6^A methylation plays crucial roles on the regulation of metabolic diseases, including obesity, type 2 diabetes, non-alcoholic fatty liver disease, hypertension, osteoporosis and immune-related metabolic diseases

*Refs* references

## m^6^A writers, erasers, readers

**Fig. 1:**
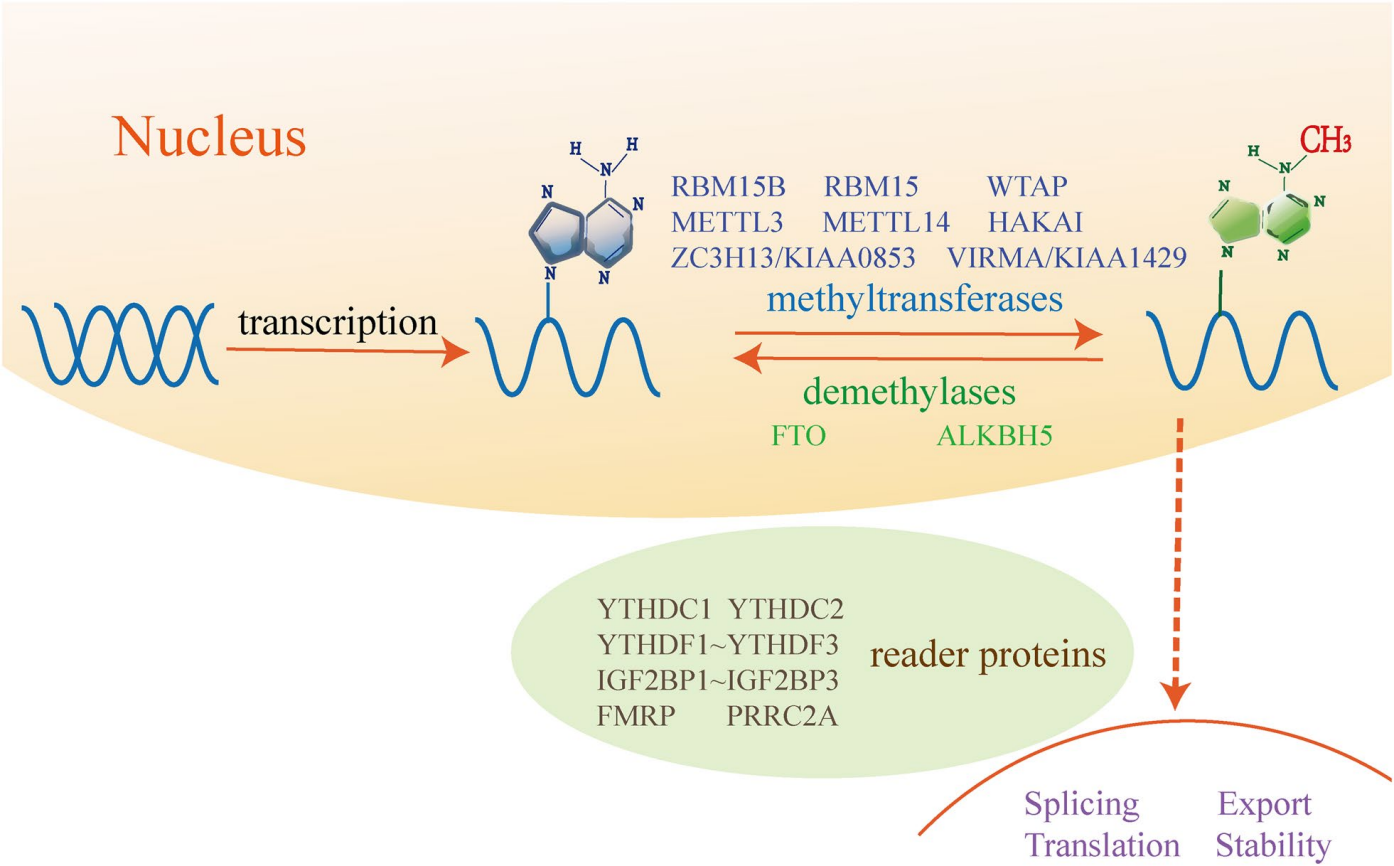
The dynamic and reversible processes of m^6^A methylation and its biological functions. m^6^A RNA modification is a widespread and reversible process which is catalyzed by “writers”, consisting of METTL3, METTL14, WTAP, HAKAI, ZC3H13/KIAA0853, VIRMA/KIAA1429, RBM15B and RBM15. Meanwhile, the m^6^A methylation can be removed by m^6^A “erasers”, including FTO and ALKBH5. Besides, it is recognized by “readers”-YTHDF1, YTHDF2, YTHDF3, YTHDC1, YTHDC2, IGF2BP1, IGF2BP2, IGF2BP3, FMRP and PRRC2A. The biological functions of m^6^A methylation on stability, translation, splicing or nuclear export are highly involved in m^6^A methylation associated diseases

The regulators in m^6^A modification are categorized as ‘writers’ and ‘erasers’ (methylation and de-methylation, respectively) and ‘readers’ (recognition) [35–37] which were presented in Fig. 1. The m^6^A methylation begins to be installed by a large multiprotein writer complex, which includes the core METTL3 and METTL14 methyltransferase subunits and many other associated regulatory subunits [38]. METTL3 is a significant catalytic component [38, 39], and METTL14 as a homolog of METTL3 shares 43% identity with METTL3, which can help their RNA substrates recognize each other [39, 40]. These two proteins can form a stable heterodimer core METTL3–METTL14 complex that acts on the cellular m^6^A deposition of nuclear RNAs and increases the methyltransferase activities in mammals [38]. Meanwhile, Wilms’ tumor 1-associated protein (WTAP), Virilizer like m^6^A methyltransferase associated protein (VIRMA/KIAA1429), an E3 ubiquitin ligase for the E-cadherin complex (HAKAI), and zinc finger CCCH-type containing 13 (ZC3H13/KIAA0853) are adaptor proteins which may guide the METTL3–METTL14 heterodimer to its target mRNAs. Besides, RNA-binding protein 15 (RBM15) and RBM15B may participate in determining which sites can be methylated [9, 41–51].

The demethylated process of m^6^A ‘erasers’ are dominated by two members of the a-ketoglutarate-dependent dioxygenase protein family, including FTO and ALKBH5 [10, 11]. ALKBH5 and FTO as powerful m^6^A demethylases can effectively demethylate m^6^A_m_ and m^6^A, but the demethylation capacity of FTO is stronger than ALKBH5 [37, 52]. FTO is a significant fat mass and obesity associated gene with a full length of 400 kp, including nine exons, which mainly locates in the 16q12-q24 of the human chromosome [53]. It is currently recognized as the most robust predictor of polygenic obesity [53, 54] as its capability of encoding for several important energy regulating proteins [55–58].

‘Readers’, YT521-B homology (YTH) family proteins, contain a YTH domain that can specifically recognize m^6^A methylation. YTHDF1, YTHDF2, YTHDF3, and YTHDC2 are predominantly located in the cytoplasm, while YTHDC1 is mainly found in the nucleus [12, 35, 59–62]. Among them, YTHDF1, YTHDC2 can recognize and bind to the methyl tag on the RNA and influence the translation of the target RNA [60]. YTHDF2 can alter the distribution of various m^6^A-containing mRNAs in the cytoplasm and affect the stability of the target RNA [60]. A newly identified m^6^A reader family including insulin like growth factor 2 mRNA binding protein 1 (IGF2BP1), IGF2BP2 and IGF2BP3 can regulate gene expression by enhancing the stability of its target RNA [63]. In addition, fragile X mental retardation protein (FMRP) has showed to promote nuclear export of methylated mRNA targets during neural differentiation by reading m^6^A [64]. Another novel m^6^A reader, proline rich coiled-coil 2A (PRRC2A), controls myelination and oligodendrocyte specification by stabilizing target mRNA [65].

## m^6^A methylation and T2D

The global prevalence of diabetes in adults is about 8% and it may increase to 10% by 2040 [66]. More than 90% of diabetes is T2D, which is characterized by hyperglycemia and dyslipidemia. Recent released studies have suggested that the m^6^A modification may play a critical role in the regulation of T2D [32, 67, 68]. For example, m^6^A highly stimulates glucose oxidation in rat adipocytes, which indicates that the proper level of m^6^A may be required to maintain certain concentration of blood glucose [67]. Many studies demonstrate that the content of m^6^A is negatively associated with the risk of T2D, as a significant reduction of m^6^A contents can been found in T2D patients [32], while, the increased mRNA expression of demethylase FTO is responsible for the reduction of m^6^A content, which may induce the complications of T2D, including obesity, cardiovascular diseases [68]. Meanwhile, high glucose stimulation contributes to the increase of FTO expression [32], and then further promotes the mRNA expression of forkhead box O1 (FOXO1), glucose-6-phosphatase catalytic subunit (G6PC), and diacylglycerol *O*-acyltransferase 2 (DGAT2) to participate in glucose and lipid metabolism [32]. Intriguingly, the levels of m^6^A methyltransferases (METTL3, METTL14, WTAP) mRNA expression are also significantly elevated in patients with T2D, but the expression of METTL3, METTL14, and KIAA1429 are negatively correlated with m^6^A content [32]. In addition, METTL3 inhibits hepatic insulin sensitivity via *N*6-methylation of FASN (fatty acid synthetase) mRNA and promoting fatty acid metabolism, which eventually results in the development of T2D [69]. In addition, METTL14 is essential for β-survival, differentiation and insulin secretion, the deficiency of METTL14 in β-cells increases cell death, changes cell differentiation and decreases β-cell mass and insulin secretion, leading to glucose intolerance and T2D [70]. Furthermore, the increased expression of m^6^A methylation upregulates the insulin/insulin-like growth factor 1 (IGF1)–AKT-pancreatic and duodenal homeobox 1 (PDX1) pathway by targeting METTL14 or METTL3 in human β-cells, which ultimately inhibits cell-cycle arrest and protects insulin secretion [71]. Besides, single nucleotide polymorphisms (SNPs) in FTO are also strongly associated with T2D, such as variant rs9939609 and rs17817449 of FTO gene [72], which are important for the development of insulin resistance and occurrence of T2D [73]. Together, m^6^A modulators might be potential therapeutic targets for maintaining glucose metabolism and preserving β-cell survival and insulin functions in T2D.

## m^6^A methylation and obesity

Obesity is an increasing risk for its related chronic diseases like NAFLD, cardiovascular diseases, diabetes and cancers [74, 75]. Obesity or adipogenesis is usually characterized by increased cell size (hypertrophy) and fat cell numbers (hyperplasia) at the cellular level. Studies have suggested that FTO-mediated m^6^A demethylation is closely related with the upregulated ghrelin production, adipogenesis, fat mass and body weight [33, 76–79]. People with a high body mass index may commonly carry FTO risk alleles [80–82] and there are some SNPs of FTO positively associated with obesity. For instance, FTO (rs17817449) is positively correlated with obesity and plasma insulin, insulin resistance, percentage body fat and fat mass in a north Indian population [83]. FTO (rs3751812) can promote obesity by altering fat deposition and disturbing serum lipid profile [84]. FTO (rs9939609 T/A) is related to increased FTO expression, reduced m^6^A ghrelin mRNA methylation, and finally results in increased energy intake and obesity by upregulating the ghrelin expression [85]. m^6^A demethylase FTO can promote adipogenesis by inhibiting the Wnt/β-catenin signaling pathway in porcine intramuscular pre-adipocytes [86]. The knockdown of FTO decreases the expression of ATG5 (autophagy-related 5) and ATG7, leading to attenuation of autophagosome formation, thereby inhibiting autophagy and adipogenesis. Meanwhile, YTHDF2 decreases protein expression of ATG5 and ATG7 by shortening the lifespan of their m^6^A-modified mRNAs [87]. Furthermore, the effect of FTO on adipogenesis also appears to be regulated via enhanced expression of the pro-adipogenic short isoform of Runt-related transcription factor 1 (RUNX1), which can promote adipocyte proliferation [77]. In the contrast, WTAP, METTL3, METTL14 are negatively related with adipogenesis by promoting cell cycle transition in mitotic clonal expansion [88, 89]. Moreover, m^6^A-YTHDF2-FTO signaling way might be crucial for the development of obesity, m^6^A—binding protein YTHDF2 can methylate mRNAs of cyclin A2 (CCNA2) and cyclin dependent kinase 2 (CDK2), and then reduce their protein expression to prolong cell cycle progression and suppress adipogenesis [90]. The methylation effect of FTO on CCNA2 and CDK2 can be reversed by epigallocatechin gallate induced YTHDF2 expression [91]. The expression of METTL3 increases via the depletion of ZFP217 (zinc finger protein 217), reversely, METTL3 knockdown rescues the siZFP217-inhibited mitotic clonal expansion and promotes CCND1 (cyclin D1). Meanwhile, YTHDF2 recognizes and degrades the methylated CCND1 mRNA, leading to the downregulation of CCND1. Consequently, cell cycle progression is blocked, and adipogenesis is inhibited [92]. Taken together, m^6^A modification may be a novel potential biomarker of obesity.

## m^6^A methylation and NAFLD

NAFLD is the most common cause of chronic liver disease among children and adults all over the world [93–95], which is characterized by steatosis, ballooning degeneration, and fatty retention of liver parenchyma cells with no history of excessive alcohol intake or other known liver disease [96]. The pathological character of NAFLD is caused by metabolic dysregulation of de novo lipogenesis, fatty acid uptake, fatty acid oxidation, and triglycerides export [97, 98]. Previous studies have found that m^6^A alteration is highly related to the development of NAFLD [34, 99, 100]. The level of FTO is elevated in hepatic tissue at NAFLD patients with hyperglycemic and hyper-insulinemic [34], which can down-regulate mitochondrial content and up-regulate triglyceride (TG) deposition, while FTO (R316A) mutant lacking demethylation activity and could not regulate mitochondria and TG content. These indicate that FTO can affect mitochondrial content and fat metabolism by modulating m^6^A levels in hepatocytes [101]. In addition, the activation of phosphatidylinositol 3-kinase (PI3K)/AKT signaling pathway may improve the development of NAFLD by suppressing FTO mediated hepatocyte regeneration [102]. Enhanced FTO expression can increase expression of lipogenic genes, containing fatty acid synthase (FASN), stearoyl-CoA desaturase (SCD) and monoacylglycerol *O*-acyltransferase 1 (MOGAT1), and intracellular TG level in HepG2 cells [101], which finally promotes hepatic fat accumulation. Meanwhile, these effects can be effectively reversed by betaine (a methyl donor) [101, 103]. Increased FTO levels are also highly involved in hepatic oxidative stress and lipid deposition which participate in the process of NAFLD [99]. Currently, dietary curcumin can affect the expression of METTL3, METTL14, ALKBH5, FTO, and YTHDF2 mRNAs, and finally improve lipopolysaccharide-induced liver injury and hepatic lipid metabolism disruption by increasing m^6^A methylation level in the liver of piglets [104]. In addition, the knockdown of METTL3 or YTHDF2 can increase the lifetime and expression of peroxisome proliferator activated receptor alpha (PPARα) mRNA, resulting in a reduction of lipid accumulation [105]. In summary, m^6^A modulators have potentials in the therapeutic function of NAFLD.

## m^6^A methylation in hypertension and cardiovascular diseases

Recent studies show that m^6^A modification is closely related to blood pressure (BP) and cardiovascular diseases [106]. For example, the m^6^A-SNP (Lys67Arg, rs197922) in golgi SNAP receptor complex member 2 gene is positively associated with hypertension in white individuals [107]. In addition, the m^6^A-SNPs (Arg389Gly, rs1801253; Ser49Gly, rs1801253) can develop hypertension as they can encode β1-adrenoreceptor, a hypertension-susceptibility candidate gene [108, 109]. rs9847953 and rs197922 have regulatory potentials to alter BP related gene expression, mRNA stability and homeostasis [110]. The m^6^A RNA modifications also involve in various mechanisms of cardiovascular diseases. For example, FTO overexpression in mouse models of myocardial infarction decreases fibrosis and enhanced angiogenesis [111]. In addition, cardiac growth is controlled by METTL3, which drives cardiomyocyte hypertrophy by catalyzing methylation of m^6^A on certain subsets of mRNAs. Whereas, diminished METTL3 promotes eccentric cardiomyocyte remodeling and dysfunction [112]. Moreover, METTL3 upregulation inhibits cellular autophagic flux and promotes apoptosis in hypoxia/reoxygenation-treated cardiomyocytes [113]. In summary, targeting m^6^A through its relative enzymes may be used as a potential diagnostic or a novel therapeutic strategy for hypertension and cardiovascular diseases in the future.

## m^6^A methylation and osteoporosis

Osteoporosis is one of the most significant bone metabolic diseases, especially aged-related osteoporosis. The low bone mass and excessive accumulation of adipose tissue in bone marrow milieu can result in architectural deterioration of the skeleton, the decrease of bone strength and an increased risk of fragility fractures [114, 115]. Recent released studies has suggested that m^6^A modification and its regulatory enzymes such as FTO, METTL3 are the key factors for osteoporosis [116–118]. The deletion of METTL3 in porcine bone marrow stem cells could promote adipogenesis and adipogenic differentiation via janus kinase 1 (JAK1)/signal transducer and activator of transcription 5 (STAT5)/CCAAT/enhancer binding protein β (C/EBPβ) pathway [119]. Also, the deletion of METTL3 in bone marrow mesenchymal stem cells disrupts cell fate and promotes osteoporosis pathological phenotypes (decreasing bone mass with incompetent osteogenic potential and increasing marrow adiposity with enhanced adipogenic potential) by reducing m^6^A methylation level in mice via parathyroid hormone (PTH)/parathyroid hormone 1 receptor (PTH1R) signaling axis [118]. In addition, the abundance of FTO can promote the differentiation of adipocyte and osteoblast from bone marrow mesenchymal stem cells by growth differentiation factor 11 (GDF11) and peroxisome proliferator-activated receptor gamma (PPARγ) in a C/EBPα-dependent manner [116]. Interestingly, FTO expression in the bone is up-regulated during aging and osteoporosis, while the expression of METTL3 is not affected by age [116]. In the contrast, FTO in osteoblasts can enhance the stability of mRNAs which protect osteoblasts from genotoxic damage through Hspa1a–NF-κB signaling way [120]. Besides, bone mineral density-associated m^6^A-SNPs may also play significant roles in the pathology of osteoporosis, including m^6^A-SNP rs17787930. rs1110720 and rs11614913 [117]. All in all, the levels of m^6^A methylation or regulators are strongly associated with osteoporosis.

## m^6^A methylation and immune-related MDs

The interactions between immune and metabolic responses play an important role in pathological development and chronic inflammation [121], including insulin resistance, insulin unresponsiveness, hepatic fat deposition and excessive adipose tissue development [122]. m^6^A methylation emerges as an significant role in immune-related MDs, for example, ALKBH5 is highly up-expressed in organs enriched in immune cells with frequent immune reactions, including thymus, spleen and thyroid [10, 123]. Also, METTL3-mediated m^6^A of CD40, CD80 and toll-like receptors 4 (TLR4) signaling adaptor TIR domain containing adaptor protein (TIRAP) transcripts enhance their translation in dendritic cells for stimulating T cell activation and the development of T lymphocytes in the thymus [124, 125]. Furthermore, the deletion of METTL3 in mouse T cells disrupts T cell homeostasis and differentiation by targeting the interleukin 7 (IL-7)/STAT5/cytokine inducible SH2 containing protein (SOCS) pathways [126]. In addition, m^6^A modification prevents TLRs activation upon binding of native mRNAs such as mRNAs with m^5^C, 5-methyluridine, 2-thiouridine substrate, m^6^A, which cannot active TLR3, TLR7 or TLR8, while unmodified RNA could activate all these human TLRs [127]. Thus, the study of m^6^A methylation on immune response may provide a new insight for the treatment of immune-related MDs, and more related mechanisms need to be clarified.

## Conclusions and perspectives

m^6^A modification is highly involved in RNA stability, localization, turnover and translation efficiency, which is crucial for the biological functions [128]. The mRNA m^6^A methylation has a wide range of effects on MDs. The researches can be conducted by many experimental methods such as m^6^A-seq (m^6^A-specific methylated RNA immunoprecipitation with next-generation sequencing), PA-m^6^A-seq (photo-crosslinking-assisted m^6^A-sequencing), and LC–MS/MS (liquid chromatography linked to tandem mass spectrometry) [4, 129, 130]. Apart from the expensive experimental screening of m^6^A sites in RNAs, some bioinformatics tools have been developed for large-scale identification of m^6^A modification sites, including SCARLET (site-specific cleavage and radioactive-labeling followed by ligation-assisted extraction and thin-layer chromatography), TargetM6A, RNA-methylPred, iRNA-Methyl and pRNAm-PC [131–135]. This m^6^A related regulatory system will promote targeted therapy for MDs.

Strategies for m^6^A-targeted drugs design are on the following: Firstly, virtual screening can be used to discover the potential compounds for experimental validation by using the drug-like SPECS database which contains about 100,000 compounds [136]; Secondly, the mechanistic study and kinetics analysis can be used to select the best m^6^A inhibitor or methyl donor [136]; In addition, differential scanning fluorometry- and liquid chromatography-based assays are applied to screen related compounds [55]; Furthermore, we can also synthetize m^6^A related compounds by utilizing a modular approach [137].

Currently, several promising agents may have potentials to treat MDs by targeting m^6^A, such as m^6^A inhibitors. It is known that FTO negatively regulated m^6^A levels and positively regulated adipogenesis, thus we can use FTO inhibitors (rhein, radicicol, epigallocatechin gallate, entacapone and meclofenamic acid) [91, 136, 138–140] to remove the potential effect of FTO. In addition, ALKBH5 is positively related to the frequent immune reactions [123], if we rule out the effects of ALKBH5 on immune cells via using ALKBH5 inhibitor (IOX3) [141], the immune-related MDs will be improved. Also, cycloleucine (a methylation inhibitor), *S*-adenosylhomocysteine (a competitive inhibitor for some adenosylmethionine-dependent methyltransferases) can be applied to downregulate m^6^A methylation directly [88, 101, 142]. In the contrast, many m^6^A regulators are useful for the improvement of MDs, for instance, METTL3, METTL14, YTHDF2 are negatively correlated with adipogenesis [87, 89]. Therefore, betaine, a methyl donor [88, 101], could be employed to upregulate m^6^A methylation directly. All in all, it’s still a long journey for the special m^6^A-targeted drugs for MDs, but the development and application of more m^6^A inhibitors or methyl donors will provide important clues to the development of m^6^A special drugs for MDs.

So far, the studies on mRNA m^6^A methylation remain poorly understood. For example, almost all the known demethylases belong to the AlkB family, and whether other proteins in or out the AlkB family are also involved in mRNA demethylation needs to be further studied. Variations in methylated and demethylated genes need to be further explored. The functions of m^6^A modification on non-coding RNAs, such as miRNA, circRNA, piRNA and lncRNA need to be unveiled in the metabolic processing. Accordingly, m^6^A—as one of the abundant basic modifications of circRNAs, lncRNA and miRNA [143–145], may have a promising future in early diagnosis on MDs through identifying downregulated or upregulated m^6^A methylation levels or mediators levels. The RNA m^6^A methyltransferases and demethylases can selectively methylate or demethylate the MDs-related genes [146, 147]. The immune cell responses play an important role in the pathological development of MDs, however, the roles of m^6^A modifications in immune-related MDs are poorly understood. Based on the functions of m^6^A modifications in immune responses, thus we speculated that m^6^A modifications in immune-related MDs might be important.

There are many problems in the m^6^A dominated diagnosis and therapies of MDs. Firstly, the biological functions of m^6^A modification in MDs needs to be further clarified. Secondly, the functions of m^6^A modification on risk factors of MDs such as aging, infection and cancers are still a tip of the iceberg. Finally, the m^6^A related treatment of MDs merely focus on FTO inhibitors, so the novel therapeutics targeting m^6^A related potents and specific small-molecule m^6^A modification inhibitors need to be further identified or developed through small-molecule compound library screening or chemical synthesis.

## Data Availability

All data reviewed and described is either included in this manuscript or available online in the relevant publications.

## Abbreviations

m^6^A: *N*(6)-Methyladenosine
m^5^C: 5-Methylcytosine
UTR: Untranslated terminal region
METTL3: m^6^A methyltransferase-like 3
FTO: Fat mass and obesity-associated protein
ALKBH5: alkB homolog 5
MDs: Metabolic diseases
T2D: Type 2 diabetes
NAFLD: Non-alcoholic fatty liver disease
WTAP: Wilms’ tumor 1-associated protein
VIRMA/KIAA1429: Virilizer like m^6^A methyltransferase associated protein
HAKAI: An E3 ubiquitin ligase for the E-cadherin complex
ZC3H13/KIAA0853: Zinc finger CCCH-type containing 13
RBM15: RNA-binding protein 15
YTH: YT521-B homology
IGF2BP1: Insulin like growth factor 2 mRNA binding protein 1
FMRP: Fragile X mental retardation protein
PRRC2A: Proline rich coiled-coil 2A
FOXO1: Forkhead box O1
G6PC: Glucose-6-phosphatase catalytic subunit
DGAT2: Diacylglycerol *O*-acyltransferase 2
FASN: Fatty acid synthetase
IGF1: Insulin-like growth factor 1
PDX1: Pancreatic and duodenal homeobox 1
SNPs: Single nucleotide polymorphisms
ATG5: Autophagy-related 5
RUNX1: Runt-related transcription factor 1
CCNA2: Cyclin A2
CDK2: Cyclin dependent kinase 2
ZFP217: Zinc finger protein 217
CCND1: Cyclin D1
TG: Triglyceride
PI3K: Phosphatidylinositol 3-kinase
FASN: Fatty acid synthase
SCD: Stearoyl-CoA desaturase
MOGAT1: Monoacylglycerol *O*-acyltransferase 1
PPARA: Peroxisome proliferator activated receptor alpha
BP: Blood pressure
JAK1: Janus kinase 1
STAT5: Signal transducer and activator of transcription 5
C/EBPβ: CCAAT/enhancer binding protein β
PTH: Parathyroid hormone
PTH1R: Parathyroid hormone 1 receptor
GDF11: Growth differentiation factor 11
PPARγ: Peroxisome proliferator-activated receptor gamma
TLR4: Toll-like receptors 4
TIRAP: TIR domain containing adaptor protein
IL-7: Interleukin 7
SOCS: Cytokine inducible SH2 containing protein
m^6^A-seq: m^6^A-specific methylated RNA immunoprecipitation with next-generation sequencing
PA-m^6^A-seq: Photo-crosslinking-assisted m^6^A-sequencing
LC–MS/MS: Liquid chromatography linked to tandem mass spectrometry
SCARLET: Site-specific cleavage and radioactive-labeling followed by ligation-assisted extraction and thin-layer chromatography
